# Idiopathic non-cirrhotic portal hypertension: a review

**DOI:** 10.1186/s13023-015-0288-8

**Published:** 2015-05-30

**Authors:** Jeoffrey NL Schouten, Joanne Verheij, Susana Seijo

**Affiliations:** Department of Gastroenterology, University Hospital Ghent, De Pintelaan 185, Ghent, Belgium; Department of Pathology, Academic Medical Center, University of Amsterdam, Amsterdam, The Netherlands; Department of Medicine, CTO, Icahn School of Medicine at Mount Sinai, New York, NY 10029 USA

**Keywords:** Non-cirrhotic portal hypertension, Portal hypertension, Variceal bleeding, Portal vein thrombosis

## Abstract

Idiopathic non-cirrhotic portal hypertension (INCPH) is a rare disease characterized of intrahepatic portal hypertension in the absence of cirrhosis or other causes of liver disease and splanchnic venous thrombosis. The etiology of INCPH can be classified in five categories: 1) immunological disorders (i.e. association with common variable immunodeficiency syndrome, connective tissue diseases, Crohn’s disease, etc.), 2) chronic infections, 3) exposure to medications or toxins (e.g. azathioprine, 6- thioguanine, arsenic), 4) genetic predisposition (i.e. familial aggregation and association with Adams-Oliver syndrome and Turner disease) and 5) prothrombotic conditions (e.g. inherited thrombophilias myeloproliferative neoplasm antiphospholipid syndrome). Roughly, INCPH diagnosis is based on clinical criteria and the formal exclusion of any other causes of portal hypertension. A formal diagnosis is based on the following criteria: 1) presence of unequivocal signs of portal hypertension, 2) absence of cirrhosis, advanced fibrosis or other causes of chronic liver diseases, and 3) absence of thrombosis of the hepatic veins or of the portal vein at imaging. Patients with INCPH usually present with signs or symptoms of portal hypertension such as gastro-esophageal varices, variceal bleeding or splenomegaly. Ascites and/or liver failure can occur in the context of precipitating factors. The development of portal vein thrombosis is common. Survival is manly limited by concomitant disorders. Currently, treatment of INCPH relies on the prevention of complications related to portal hypertension, following current guidelines of cirrhotic portal hypertension. No treatment has been studied aimed to modify the natural history of the disease. Anticoagulation therapy can be considered in patients who develop portal vein thrombosis.

## Introduction

Idiopathic non-cirrhotic portal hypertension (INCPH) is a rare disease characterized by of intrahepatic portal hypertension in the absence of cirrhosis, other causes of liver disease and splanchnic venous thrombosis [1–7]. Histological features of INCPH comprise a wide spectrum of nonspecific features, ranging from minor changes, sinusoidal dilatation, phlebosclerosis and portal fibrosis to nodular regenerative hyperplasia. It is still unclear whether this repertoire of histological changes reflects different stages of the disease, or it could also be that INCPH comprises different nosologic entities that share the same clinical presentation. Different conditions have been associated to this disorder including immune-based diseases, recurrent infections, HIV infection and antiretroviral treatment, trace metals, certain medications and prothrombotic factors [1, 3, 7–9]. The pathophysiological mechanisms causing INCPH remain largely unknown. Patients with INCPH usually present with signs and symptoms of portal hypertension (PH) such as splenomegaly, thrombocytopenia and variceal bleeding [1, 2, 5]. Patients can develop additional liver-related complications such as ascites, hepatic encephalopathy, portal vein thrombosis (PVT) or liver failure, that could eventually require liver transplantation (LT) [2, 3, 6, 10, 11].

Orpha number: ORPHA64743

### Epidemiology

Although INCPH has a worldwide distribution, it is particularly prevalent in Asia [4, 12, 13]. It is more frequent in socioeconomically disadvantaged individuals. Improvements in hygiene and living standards may explain the decreasing incidence of INCPH in Japan during the last decades, and the low prevalence of the disease in Western countries [7, 9, 14, 15]. Gender and age disparities have also been reported [4, 15]. In Western populations, median age at diagnosis is 40 years, with predominance in male gender. Conversely, Asian patients tend to be diagnosed at a younger age. In summary, differences in socioeconomic status, living conditions, pathogen exposure and ethnicity may play a role in INCPH development.

### Etiology and pathophysiology

#### Etiology

The etiology of INCPH is unknown [1, 4, 9, 14, 16]. Strikingly, small series and case studies show its association with an array of rare disorders; whether these associations are more than fortuitous remains unclear. Roughly, the potential mechanisms involved in INCPH pathogenesis can be classified in five main categories: immunological disorders, chronic infections, exposure to medications or toxins, genetic disorders and prothrombotic conditions (Table 1). A combination of these factors is also very likely. Associated conditions in 4 recently reported European series are described in Table 2 [2, 6, 10, 11, 16].

**Table 1:** Associated disorders of idiopathic non-cirrhotic portal hypertension

Immunological disorders	Common variable immunodeficiency syndrome [22, 54]
	Connective tissue diseases [55]
	Crohn’s disease [26, 27]
	Solid organ transplant [56, 57]
Infections	Bacterial intestinal infections [21]
	Human immunodeficiency virus (HIV) infection [2, 9, 14, 22]
Medications and toxins	Thiopurine derivatives (didanosine, azathioprine, cis-thioguanine) [27, 58]
	Arsenicals [28]
	Vitamin A [59]
Genetic disorders	Adams-Olivier syndrome [32]
	Turner syndrome [30]
	Phosphomannose isomerase deficiency [60]
	Familial cases [31, 61]
Prothrombotic conditions	Inherited thrombophilias [2, 6, 10, 11, 62]
	Myeloproliferative neoplasm [2, 6, 10, 11, 62]
	Antiphospholipid syndrome [2, 6, 10, 11, 62]

**Table 2:** Associated conditions in 4 recently reported series of European patients with idiopathic non-cirrhotic portal hypertension

Reference	Hillaire et al. [6]	Cazals-Hatem et al. [11]	Schouten et al. [10]	Siramolpiwat et al. [2]
Prothrombotic disorder	6 PS deficiency	3 PS deficiency	3 PS deficiency	1 PS deficiency
	2 PC deficiency	3 PC deficiency	3 PC deficiency	2 FII Leiden
		2 MTHFR mutation	1 MTHFR mutation	1 FV Leiden
		3 FII Leiden	2 FV Leiden	
Haematological malignancy	1	0	4	5
Myeloproliferative neoplasm	6	10	3	0
Chronic HIV infection	0	0	5	15
Autoimmune disorder	3	10	1	9
Crohn’s disease	0	0	3	0
Genetic disorder	0	0	4	0
Solid organ malignancy	0	0	1	0
Azathioprine treatment	0	0	8	0
Arsenicals	-	0	4	0
No associated conditions	12	31	26	30
Incomplete evaluation	2	-	-	9
Total^a^	28	59	62	69

*FII* factor II, *FV Leiden* factor V Leiden, *HIV* human immunodeficiency virus, *MTHFR* metilentetrahydorfolate reductase, *PC* protein C, *PS* protein S

(^a^): Some patients have more than one associated condition

#### Immunological disorders

In Western countries, INCPH is frequently associated with immunological disorders [3, 5, 7, 17, 18]. In patients with systemic sclerosis, enhanced fibrogenesis seems to have a major pathogenic role [8]. Also, in patients with systemic lupus erythematosus, immunoglobulin interference with prostacyclin formation has been found to increase microthrombosis vulnerability [19]. In patients with celiac disease, an elevation of IgA anticardiolipin antibodies could be responsible for the obliteration of small vessels [1–7]. Interestingly, 70 % of patients with primary hypogammaglobulinemia have histological features of INCPH [1, 3, 7–9, 20].

#### Chronic infections

Data indicate that intestinal infection with *E. coli* might cause recurrent septic embolization and subsequent obstruction of small portal veins, a probable trigger of INCPH. The high prevalence of INCPH in low socioeconomic areas with a high rate of abdominal infections in early childhood lends credit to this theory [1, 2, 4, 5]. In addition, experimental studies demonstrate how *E. coli* injection into the portal vein results in the development clinical and histological characteristics of INCPH [2, 3, 6, 10, 11, 21].

In Western countries, INCPH has been reported increasingly in patients with human immunodeficiency virus (HIV) infection [4, 9, 12–14, 17, 22–24]. Prolonged monotherapy or short-term combination treatment with didanosine and stavudine are independent risk factors for the development of this disorder, suggesting a potential role for mitochondrial toxicity in the development of INCPH [7, 9, 14, 15, 17, 22]. A recent multicenter study demonstrated a genetic predisposition to develop INCPH in HIV infected patients chronically exposed to didanosine [4, 15, 25]. Despite these data, it is difficult to assign a definitive etiopathogenic role of didanosine, as the drug has been widely used for the treatment of HIV in the past. Alternatively, a high prevalence of pre-existing hypercoagulability, mainly due to protein S deficiency, possibly leading to vascular obstruction, has also been reported in patients with HIV-related INCPH [1, 4, 9, 14, 16]. Hence, additional data will be needed to define a causal role of didanosine exposure and HIV-associated INCPH [2, 6, 7, 10, 11, 16, 17].

#### Exposure to medication and toxins

Besides didanosine, exposure to other medications and chemicals has been reported to be associated with INCPH. Azathioprine, 6-thioguanine and arsenic as Fowler’s solution are the most frequently reported drugs linked with this disorder [3, 5, 7, 17, 26–28]. Although it is tempting to blame drug intake and chemical exposure as primary etiological factors, only a very small proportion of the patients treated with the above mentioned drugs or exposed to these chemicals develop clinical or histological signs of INCPH. Clearly, additional factors may play a pathogenic role in these patients.

#### Genetic disorders

Reports on familial aggregation of INCPH and presence of its key histologic features in congenital disorders (e.g. Adams-Oliver syndrome, Turner disease) indicate a possible genetic component in INCPH [18, 29–33]. There is evidence of an association between HLA-DR3 and INCPH, what also supports an immunogenetic basis of this disorder [8, 31].

#### Prothrombotic conditions

Thrombophilia seems invariable behind some clinical and histologic features of patients with INCPH. There are different pieces of evidence suggestive of this including the high prevalence (30-50 %) of pre-existing hypercoagulability commonly reported in INCPH patients [2, 6, 10, 11, 19, 34]. Also, these patients tend to have a relatively high incidence of portal vein thrombosis [2, 6, 10, 11]. In addition, certain pathologic features in liver specimens of INCPH patients support a dominant role of thrombophilia in the development of INCPH [6, 11, 35, 36]. Presence of obliterative portal venopathy (i.e. luminal narrowing or obliteration of small portal venous branches accompanied by dense deposits of elastic fibers) in liver specimens of INCPH patients is highly suggestive of previous thrombotic episodes. Furthermore, the majority of liver explants from transplanted INCPH patients demonstrate organized old thrombi in the large portal vein branches [37, 38].

### Pathogenesis

A dual theory, implicating both intrahepatic vascular obstruction and increased splanchnic blood flow, has been suggested to explain portal hypertension in INCPH patients [1, 6, 39]. An increased intrahepatic resistance likely results from the obstructed intrahepatic vessels (i.e. phlebosclerosis) and distorted intrahepatic angioarchitecture (i.e. nodular regeneration). The mechanisms responsible for the obliteration of portal venules remain unknown. Several hypotheses have been proposed [1, 34, 39], including aberrant coagulation activation or thrombosis, acquired or inherited disorders of vascular remodeling, and endothelial injury from immune cells [40]. Similar to cirrhosis, the imbalance of different vasoactive mediators causing intrahepatic vasoconstriction could also be considered. Additionally to the increased intrahepatic resistance, a portal venous overflow secondary to splenomegaly has been linked to the development of portal hypertension in INCPH patients [41, 42]. Overproduction of nitric oxide, released in the sinus lining spleen cells, could also justify the dilatation of splenic sinuses and subsequent massive splenomegaly frequently found in INCPH patients [1, 43].

### Clinical manifestations

Complications related to portal hypertension dominate the signs and symptoms present in patients with INCPH [1, 2, 34, 39]. The liver function is usually preserved. Variceal bleeding is the most common clinical feature. Unlike cirrhotic patients, prognosis of variceal bleeding in INCPH is usually good due to the preserved liver function. In those patients without variceal bleeding at diagnosis, over 75 % had varices at the initial endoscopy [2, 34]. A recent study has shown that the 1-year probability of developing small and large varices was 10 % and 13 %, respectively; this is similar to what is described in cirrhotic patients [2]. This study also showed that in patients with large varices, the 1-year probability of first bleeding episode despite primary prophylaxis was 9 %. In addition, the 1-year probability of re-bleeding despite combined secondary prophylaxis (i.e. beta-blockers and endoscopic band ligation) was 22 % [2].

Ascites is reported in up to 50 % of cases, and it usually develops in the context of precipitating factors such as variceal bleeding or infections. Generally, it is easily controlled with low dose of diuretics and resolution of the trigger [1, 10]. Hepatic encephalopathy is a rare complication and it is also related to precipitating factors. There are anecdotic reports of hepatopulmonary syndrome, portopulmonary hypertension and hepatocellular carcinoma. Over 95 % of patients have splenomegaly and it can cause left upper quadrant’s abdominal pain.

Portal vein thrombosis (PVT) is also common, with a reported prevalence that ranges from 13-46 % [2, 6, 10]. A recent study found a 9 % annual probability of developing PVT. HIV infection and the presence of variceal bleeding at diagnosis have been described as factors independently associated with a high risk of developing PVT [2, 7]. Remarkably, most patients are asymptomatic at the time of PVT diagnosis. Therefore, it may be useful to screen for the presence of PVT in INCPH patients. It is unclear, however, the optimal frequency or best imaging modality in this context.

### Diagnosis

There is a lack of a specific positive test that leads to an INCPH diagnosis. It is based on clinical criteria and the formal exclusion of other causes of PH; this represents a clinical challenge, even in experienced liver units. Consequently, INCPH is frequently unrecognized, and in many instances patients are misdiagnosed with liver cirrhosis [44, 45].

### Criteria and differential diagnosis

The diagnosis of INCPH is a diagnosis of exclusion, based on the following previously reported criteria [1]: 1) presence of unequivocal signs of portal hypertension (e.g., gastroesophageal varices, ascites, and/or splenomegaly); 2) absence of cirrhosis, advanced fibrosis or other causes of chronic liver diseases that can cause PH by appropriate serological, biochemical tests and liver biopsy and; 3) absence of thrombosis of the hepatic veins or of the portal vein at imaging studies performed at diagnosis.

Therefore, the current diagnostic work up for INCPH should include: 1) detailed medical history to investigate concomitant diseases and exposure to drugs, medications or toxins, 2) liver imaging to evaluate the patency of the splanchnic venous axis, 3) laboratory tests to rule out other causes of liver diseases and/or PH and 4) a mandatory liver biopsy to discard cirrhosis and other causes of chronic liver disease with or without PH. As a result, a diagnosis of INCPH can only be made upon the exclusion of liver cirrhosis, portal vein thrombosis, Budd-Chiari syndrome, chronic liver diseases causing noncirrhotic portal hypertension (e.g. chronic viral hepatitis, primary biliary cirrhosis, non-alcoholic steatohepatitis, alcoholic steatohepatitis and autoimmune hepatitis) and conditions causing portal hypertension (congenital liver fibrosis, sarcoidosis and schistomiasis).

#### Liver function tests

Liver function tests are usually within normal range; jaundice is rarely seen at diagnosis. Transient impairments in liver function may occur in the context of variceal bleeding or infection. Anemia, leukopenia, and thrombocytopenia are common due to hypersplenism.

#### Imaging

Comprehensive liver imaging is required before INCPH can be confidently diagnosed. The goals of liver imaging are twofold: 1) to determine the presence of radiological signs of PH such as splenomegaly, collaterals or ascites, and 2) to evaluate the patency of the hepatic veins and the porto-spleno-mesenteric venous axis. Of note, most patients also present radiological signs of chronic liver disease (i.e. liver surface nodularity) despite the lack of histologic cirrhosis [45]. Doppler ultrasound in addition to CT angiography or MRI angiography is the recommended strategy.

#### Liver biopsy / liver pathology

**Fig. 1:**
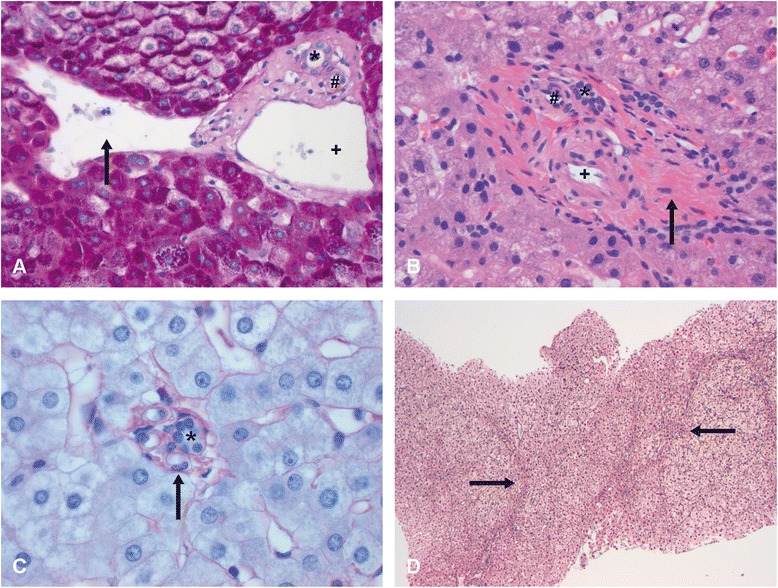
**a** Paraportal shunting vessel (arrow), herniating into the liver parenchyma. The adjacent portal tract has a bile duct (*), hepatic artery (#) and portal vein (+); PAS staining, 20x. **b** Phlebosclerosis. In a fibrotic portal tract (arrow), a bile duct (*), hepatic artery (#) and arterialised portal vein (+) are present; haematoxylin and eosin, 20x. **c** Hypoplastic portal tract in which the lumen of the bile duct (*) is smaller than the diameter of the surrounding hepatocytes; PAS-amylase staining, 40 x. **d** Nodular regenerative hyperplasia (NRH) with central hyperplasia and an atrophic rim (arrow) in the absence of fibrosis; reticulin staining, 10x

The morphological features associated with INCPH can be sometimes subtle, what makes pivotal an adequate histological evaluation by expert liver pathologists. It is essential to systematically assess the different anatomical structures in the liver, including their size, topography and morphology. At the portal tracts, presence of a bile duct, a branch of the hepatic artery and of a normally sized portal vein needs to be determined. Hypoplastic or minute portal tracts, with a lumen of the bile duct or artery smaller than the surrounding hepatocytes are typical of INCPH (Fig. 1) [35]. These are thought to be portal tract remnants, resulting from resorption of normal portal tract collagen [46]. Portal sclerosis or hepatoportal sclerosis is also a common feature. It consists of fibrous thickening of the portal vein wall and may result from portal vein thrombosis and consequent organization, either from the larger portal vein branches or in the context of local portal tract pathology. Some authors use the term obliterative portal venopathy for this entity. Other findings in the context of INCPH in the (peri)portal area include dilated portal veins, abnormal spacing between portal tracts and veins, an increased number of vascular structures in the portal tracts, arterialization of the wall of portal veins and the presence of paraportal shunting vessels and/or herniating in the liver parenchyma [38, 47, 48].

In the liver parenchyma, abnormalities that may be seen in the context of INCPH include sinusoidal dilatation, congestion and pericellular fibrosis, aberrant hepatic vessels and dilatation of the central vein with or without perivenular fibrosis. The lumen of the central veins can be (partially) occluded associated with different toxic agents. A regenerative, compensatory response can be the result of a heterogeneous blood flow in the presence of circulatory abnormalities at different levels of the microcirculation. This might lead to nodular regenerative hyperplasia, showing micronodular transformation, with central hyperplasia and an atrophic rim in the absence of fibrosis [48, 49]. Pathologists should become familiar with these features, in order to suggest or support the clinical diagnosis of INCPH in patients with non-cirrhotic portal hypertension.

#### Other investigations

INCPH is an intrahepatic presinusoidal cause of PH [44, 50]. Hepatic venous pressure gradient (HVPG) is normal (≤5 mmHg) or slightly increased (5-10 mmHg) but below the previously described cut-off for clinically significant portal hypertension in cirrhosis (CSPH; HVPG>10 mmHg) [37, 51]. Also, liver stiffness value on transient elastography (Fibroscan**®**) is lower than the described cut-off values for diagnosing cirrhosis, varices and CSPH [2, 7, 51]. Thus, lower values for HVPG and liver stiffness than those described for cirrhosis and CSPH can be helpful by ruling out cirrhosis in a patient with signs of PH.

### Treatment

#### Management of portal hypertension

Data on management and prophylaxis of variceal bleeding in INCPH patients are scarce with a remarkable lack of randomized controlled trials. There are no specific guidelines for the management of PH in patients with INCPH. Nevertheless, expert opinion recommends following the guidelines of prophylaxis and management of PH in cirrhotic patients [1]. A recent cohort study reported good long-term outcome by applying a management strategy based on current guidelines for cirrhotic variceal bleeding [2].

Briefly, primary and secondary prevention of variceal bleeding includes the use of non-selective beta-blockers and endoscopic variceal ligation. Trans-jugular intrahepatic portosystemic shunting (TIPSS) is an effective alternative in patients who fail to respond to medical and endoscopic therapy. Management of acute variceal bleeding includes early pharmacological treatment with vasoactive drugs, early endoscopic control of bleeding, careful blood product replacement, and prophylactic antibiotics [52]. Guidelines also recommend withdrawing any drug potentially associated with INCPH (e.g. azathioprine) and treating any associated medical conditions [52].

#### Liver transplant

Even though INCPH patients usually have well preserved liver function, and PH related complications are successfully controlled, some patients may require a LT. Some of the reported indications for LT include unmanageable PH, progressive liver failure, chronic hepatic encephalopathy, hepatopulmonary syndrome and hepatocellular carcinoma. Post-LT outcomes of INCPH patients are good and the disease tends not to recur. However, data on this issue are limited and mostly based on small cohorts [1, 45].

#### Management of PVT

The use of anticoagulation in the management of PVT in INCPH is controversial, mainly due to the lack of prospective data. Nevertheless, we believe that anticoagulation therapy must be considered in patients with underlying prothrombotic conditions and in patients who develop PVT. A recent retrospective series described 15 patients with INCPH and PVT that were treated with anticoagulants. At the end of follow-up, 54 % of patients achieved some degree of recanalization [2]. Regarding anticoagulation in these patients, some issues that need to be addressed: 1) which are the subgroup of PVT patients that benefit from anticoagulation, 2) which is the best anticoagulantion modality (low molecular weight heparins *vs* vitamin-K antagonist *vs* new oral anticoagulants), 3) which is the optimal duration of anticoagulation and 4) which are the early predictors of response. Another important point is whether anticoagulation may have a role in the prevention of PVT. Based on the high prevalence of thrombophilia, the frequent presence of thrombosis of small intrahepatic and main portal veins in INCPH, it would be even more important to determine whether anticoagulation could play a role to prevent disease progression.

### Prognosis

Very few studies have evaluated the long-term prognosis of INCPH patients. Overall, prognosis is generally better than in patients with cirrhosis and a similar degree of portal hypertension. As mentioned above, this may be due to the fact that most INCPH patients have well preserved liver function. However, a small subgroup of patients will develop liver failure and will require LT. Two recent European cohort studies evaluated prognosis of INCPH [2, 10, 32]. The Dutch study reported low overall and LT-free survival, 78 % and 72 % at 5 years, respectively. However, it should be noted that only 13 % of patients died from liver-related causes. Conversely, the Spanish cohort reported 86 % of LT-free survival at 5 years. Interestingly, ascites was identified as a poor prognostic factor in both studies. The presence of a concomitant severe disorder such as an immunological disease or malignancy was also identified as a poor prognostic factor in the Spanish study.

### Conclusions and future perspectives

Over the last decade, numerous efforts have tried to clarify different aspects of INCPH. First, the nomenclature concerning this clinical disorder has been ambiguous and highly depended on histological features. To facilitate future studies and subsequently enhance our understanding of the disease, common terminology and diagnostic criteria have been developed [1]. Regarding pathophysiology, some genetic traits have been identified in HIV-associated INCPH [25]. Furthermore, cohort studies performed in Europe provided new insights into the natural course and prognosis of these patients [2, 10, 11]. Despite these improvements, several uncertainties related to its pathogenesis should be further addressed. Large multicenter studies studying INCPH prevalence, associated disorders, natural course and prognosis are an unmet need. The diagnosis of INCPH still relies on clinical and histologic elements; future research should provide diagnostic and prognostic biomarkers of the disease. Furthermore, no randomized controlled trials have been yet performed. Currently, treatment of INCPH patients is based on the prevention of complications of portal hypertension as per guidelines for patients with liver cirrhosis. These treatment modalities have not been systematically evaluated in INCPH. So far, no treatment able to modify the course of the disease or to prevent complications has been tested in INCPH. Considering the role of thrombophilia in the pathophysiology of this disorder, it seems that anticoagulation therapy could prevent the progression of INCPH, but prospective controlled data are still needed. Limited available data in INCPH patients show no increased risk of serious bleeding [2, 11, 53] and a significant rate of portal vein recanalization in patients with associated portal vein thrombosis [2]. Furthermore, evidence generated in other vascular liver diseases such as PVT or Budd-Chiari syndrome demonstrate that prolonged anticoagulation improves outcomes without increasing significantly the risk of serious bleeding. Hopefully, future randomized trials will provide new tools to tackle this orphan disease and improve our understanding of its complex pathophysiology.

### Nomenclature

Idiopathic non-cirrhotic portal hypertension

non-cirrhotic portal fibrosis

hepatoportal sclerosis

incomplete septal cirrhosis

obliterative portal venopathy

partial nodular transformation

## Abbreviations

CSPH: Clinically significant portal hypertension in cirrhosis
INCPH: Idiopathic non-cirrhotic portal hypertension
HIV: Human immunodeficiency virus
LT: Liver transplant
PH: Portal hypertension
PVT: Portal vein thrombosis
TIPS: Trans-jugular intrahepatic portosystemic shunting
